# FOXO transcription factor family in cancer and metastasis

**DOI:** 10.1007/s10555-020-09883-w

**Published:** 2020-05-05

**Authors:** Yannasittha Jiramongkol, Eric W.-F. Lam

**Affiliations:** Department of Surgery and Cancer, Imperial College London, Hammersmith Hospital Campus, London, W12 0NN UK

**Keywords:** Forkhead, Transcription factor, Cancer metastasis, Tumour suppressor, Post-translational regulation, Protein interactions

## Abstract

Forkhead box O (FOXO) transcription factors regulate diverse biological processes, affecting development, metabolism, stem cell maintenance and longevity. They have also been increasingly recognised as tumour suppressors through their ability to regulate genes essential for cell proliferation, cell death, senescence, angiogenesis, cell migration and metastasis. Mechanistically, FOXO proteins serve as key connection points to allow diverse proliferative, nutrient and stress signals to converge and integrate with distinct gene networks to control cell fate, metabolism and cancer development. In consequence, deregulation of FOXO expression and function can promote genetic disorders, metabolic diseases, deregulated ageing and cancer. Metastasis is the process by which cancer cells spread from the primary tumour often *via* the bloodstream or the lymphatic system and is the major cause of cancer death. The regulation and deregulation of FOXO transcription factors occur predominantly at the post-transcriptional and post-translational levels mediated by regulatory non-coding RNAs, their interactions with other protein partners and co-factors and a combination of post-translational modifications (PTMs), including phosphorylation, acetylation, methylation and ubiquitination. This review discusses the role and regulation of FOXO proteins in tumour initiation and progression, with a particular emphasis on cancer metastasis. An understanding of how signalling networks integrate with the FOXO transcription factors to modulate their developmental, metabolic and tumour-suppressive functions in normal tissues and in cancer will offer a new perspective on tumorigenesis and metastasis, and open up therapeutic opportunities for malignant diseases.

## Introduction

Cancer is a leading cause of death worldwide. It is a group of diseases that can initiate in any tissue or organ when abnormal cells grow uncontrollably and migrate from their original sites to invade other parts of the body. Metastasis is the process by which cancer cells spread from their origins to secondary sites of the body, often *via* the bloodstream or the lymphatic system. In most cases, metastatic cancer cannot be cured by treatment. Because of this, metastasis is the major cause of cancer mortality and is responsible for over 90% of cancer deaths [1]. Forkhead box (FOX) proteins are a vast group of transcription factors united by an evolutionarily conserved winged-helix DNA binding domain. FOXOs (forkhead box proteins of class O subgroup) are considered to be tumour suppressors by virtue of their established functions in cell cycle arrest, apoptosis, senescence, differentiation, DNA damage repair and scavenging of reactive oxygen species [2]. Besides these cellular processes essential for cancer initiation (tumorigenesis), FOXOs have also emerged as key modulators of metastasis and angiogenesis, two key factors critical for cancer progression and establishment at secondary sites.

The FOX winged-helix structure, reminiscent of a butterfly, consists of three N-terminal α-helices, three β-strands and two loops [3]. Through this unique structural feature, the FOX proteins recognise the *cis*-regulatory sequences in their target genes to direct gene expression [4]. To date, 19 (A–S) subfamilies and 50 mammalian FOX proteins have been identified and are classified according to their sequence homology within the winged-helix and other functional domains. Despite the FOX proteins possessing highly analogous DNA binding domains, their distinct tissue-specific expression patterns and regulatory mechanisms provide them their dedicated functions [5]. However, in addition to their specific roles, evidence suggests that FOX proteins also possess a certain degree of functional redundancy to safeguard organisms against a broad spectrum of developmental and metabolic diseases due to the loss of function of a single-core FOX protein or gene in haploid insufficiency. In consequence, the misregulation, misexpression and/or mutation of FOX genes can lead to human genetic and metabolic diseases, deregulated ageing and cancer.

## Normal FOXO function

In mammals, the FOXO subfamily consists of four members (FOXO1, FOXO3, FOXO4 and FOXO6). These four FOXO transcription factors bind to their target genes as monomers or heterodimers to control cell fate under distinct conditions [5]. They also interact with co-activator, co-repressors and other protein partners to modulate and to fine-tune their activity. All FOXO proteins have 4 different core functional domains, namely the winged-helix DNA binding domain, the nuclear localisation sequence, the nuclear export sequence and the transactivation domain [6]. The FOXO-DNA binding domain recognises and binds to the consensus sequences (5′-GTAAA(C/T)A-3′) in the genome [7]. Furthermore, the consensus flanking sequences also play a critical part in determining the interaction specificity between the target gene and FOX protein [6]. Each of the FOXO proteins is differentially expressed in distinct tissues. For instance, FOXO1 and FOXO4 are highly expressed in adipose tissue and skeletal muscle, respectively [8]. FOXO3 is ubiquitously expressed in multiple tissues, including the brain, kidney and heart, whilst FOXO6 is predominantly expressed during the development and in nervous tissue [8, 9]. Despite their distinct tissue-specific expression patterns, regulatory overlap and functional redundancy among FOXO proteins have been observed [10]. This may impact the role and regulation of FOXOs in multiple FOXO protein–expressing cells [10].

FOXO genes are conserved throughout evolution from lower organisms to mammals but exist only as a single gene in invertebrates. FOXO is known as dFOXO in the fruit fly *Drosophila melanogaster*, Daf-16 in the nematode *Caenorhabditis elegans* and FoxO in *Hydra vulgaris* [11, 12]. In fact, the first forkhead (FOX) gene was initially identified in fruit flies as a genetic mutation to a homeotic gene, leading to the development of an abnormal forked head structure [13]. A later study showed that dFOXO controls *Drosophila* lifespan and mediates insulin signalling in flies [14]. In *C. elegans*, Daf-16 is characterised as a downstream target of insulin/insulin-like growth factor 1 (IGF-1) pathway and the expression of Daf-16 is associated with *C. elegans* ageing and longevity [15]. In *Hydra*, FoxO is a critical regulator for unlimited lifespan [16]. Whilst direct experimental evidence for a role for FOXO in longevity has not been demonstrated in mammals, studies from these evolutionarily conserved model organisms suggest that FOXO proteins are important for the regulation of development and senescence/ageing. Collectively, these FOXO transcription factors are involved in the regulation of the cell cycle, apoptosis and metabolism. In the experimental model organisms, FOXOs have also been found to control stem cell maintenance and lifespan as well as age-related diseases, such as cancer, ageing and diabetes. Multiple upstream pathways regulate FOXO activity through post-translational modifications and co-factor interactions. The diversity of this upstream regulation and the downstream effects of FOXOs suggest that they function as regulators of tissue homeostasis over time and coordinators of responses to environmental changes, including growth factor deprivation, metabolic stress (e.g. metabolite starvation) and oxidative stress [17].

As transcription factors, the predominant mode of action of FOXO proteins is through binding to FHRE elements located at proximal gene promoter regions and recruiting other components of the transcription apparatus, including other transcription factors, transcriptional co-factors and chromatin regulators to modulate target gene transcription [2]. Compacted chromatin constitutes a barrier to transcription activators accessing promoters, and studies using a recombinant FOXO1 protein have shown that FOXO proteins can operate as ‘pioneer’ factors, by recognising their cognate sites within the promoter on a nucleosome, opening up chromatin and conferring an active chromatin state for transcription to proceed [18, 19]. This role of FOXO proteins as pioneer factors is further confirmed by later studies showing that FOXO1, like other forkhead proteins, can open and remodel chromatin and recruit additional regulatory factors to promote transcription *via* its winged-helix motif [20, 21]. Moreover, recent epigenetic studies have shown that FOXO3 is also recruited to the more distal gene regulatory elements called enhancers. In these cases, FOXO3 and, probably, other FOXOs function by binding to already active enhancers to further promote their ability to drive cell type–specific gene expression [22].

## Tumour-suppressive roles of FOXOs

### FOXOs and tumorigenesis

FOXOs are considered to be tumour suppressors by virtue of their established functions in cell cycle arrest, senescence, apoptosis, differentiation, DNA damage repair and scavenging of reactive oxygen species [2]. Studies using FOXO gene knockout mice have helped to confirm FOXO proteins as genuine tumour suppressors [23]. FOXO (*foxo1/3/4*^*−/−*^) triple-knockout mice develop thymic lymphomas and haemangiomas. These triple FOXO1/3/4-deficient animals have been shown to be predisposed to lymphomagenesis through the loss of restriction on cellular proliferation and survival. At the same time, these triple-FOXO1/3/4-deficient animal studies also reveal that the FOXO isoforms have overlapping functions in distinct biological functions in specific tissues, and that FOXO proteins are associated with cancer progression, metastasis and angiogenesis. FOXO proteins have been proposed as tumour suppressors primarily because of their established functions in promoting cell cycle arrest and apoptosis as well as preventing the accumulation of damages induced by genotoxic agents and oxidative stress [2, 3, 24].

### FOXOs and senescence

Senescence is an irreversible state of cell cycle arrest and a critical tumour-suppressive barrier to obstruct neoplastic transformation of stem cells. It limits the renewal capacity of stem cells and cancer cells. A role for FOXO protein in cellular senescence is confirmed by an *in vivo* study showing that oncogene-induced senescence also involves the repression of the phosphoinositide 3-kinase (PI3K)-Akt oncogenic signalling pathway and the consequent induction of FOXO activity [25]. In support of this, FOXO3 overexpression or inhibition of the PI3K-Akt signalling axis can induce cells to enter senescence through promoting the expression of p27^Kip1^ [26]. In addition, FOXO3 promotes the expression of the retinoblastoma family protein p130 (RB2) to induce senescence in proliferating cells [26, 27]. FOXO3 can also repress the expression of the potent oncogene FOXM1 to limit stem cell renewal to trigger senescence [28–31]. FOXM1 can counteract oxidative stress–induced senescence through enhancing the transcription of the cell self-renewal Bmi-1 gene [32]. Moreover, inhibition of FOXM1 in cancer cells, such as those of breast, gastric, gallbladder and liver cancer, leads to cellular senescence [33–36]. In agreement, overexpression of the cyclin-dependent kinase (CDK)4/6-targeting microRNA miR-506 can induce senescence in ovarian cancer cells through repressing FOXM1 [37]. Likewise, the CDK4/6 inhibitor LEE011 can also induce senescence in neuroblastoma cells through restricting the induction of FOXM1 [38]. Collectively, these findings propose a key tumour-suppressive role for FOXO proteins and downstream targets in cellular senescence in both normal and cancer cells.

### FOXOs and autophagy

As tumour suppressors, FOXOs play multiple roles in restricting cancer development and progression. FOXO proteins are involved in the regulation of autophagy which functions to destroy and recycle the cytoplasmic organelles and macromolecules. Autophagy is a tumour-suppressive mechanism in that it can prevent cellular transformation by preventing the accumulation of carcinogenic defective lipids, proteins and organelles. Moreover, it is also a mediator of anticancer chemotherapy–induced cell death [39]. Conversely, autophagy also enables cancer cells to survive under stress conditions, such as nutrient starvation, oxidative stress and chemotherapy. For example, haploinsufficiency of the autophagy genes, such as LC3, BECN1 and Bif-1, has been shown to drive chromosome instability, increase migration and promote early tumorigenesis [40–43]. One of the first reports of FOXO3 having a role in autophagy comes from a mouse muscle atrophy study showing that FOXO3 activates protein degradation by inducing autophagy in skeletal muscle cells [44]. FOXO proteins are also involved in the regulation of autophagy in order to recycle essential amino acids during nutrient starvation [45]. FOXO3 has been shown to induce autophagy in a FOXO1-dependent fashion by activating PIK3CA to enhance the PI3K-Akt activity [46]. Specifically, FOXO1 depletion has been found to attenuate FOXO3-induced autophagy in a number of cell lines [46]. Autophagic cell death is enhanced when the acetylated FOXO1 binds to an E1-like autophagy-related protein 7 (Atg7), an essential initiator of autophagy [47]. Despite these findings showing that FOXOs have a tumour-suppressive role in the context of autophagy, other reports indicate they have an oncogenic role. For example, studies from neuronal cells reveal that c-Jun N-terminal kinase (JNK) deficiency causes increased autophagy by promoting cell survival through the FOXO1-BNIP3-Beclin-1 pathway [48]. In these JNK-deficient neurons, FOXO1 is activated to promote the expression of BNIP3 which displaces the autophagic effector Beclin-1 from inactive Bcl-XL complexes to induce autophagy and cell survival [48]. However, under energy stress conditions, p38 mitogen-activated protein kinase (MAPK) has also been shown to induce this FOXO-BNIP3 axis to repress mTORC1 and cell survival [49]. Similarly, the deacetylase SIRT1 has been shown to repress the ability of acetylated FOXO3 to induce BNIP3 expression to promote apoptosis and attenuate autophagy [50]. On the evidence of these findings, the role of FOXO proteins in autophagy is likely to be context-dependent and can be a double-edged sword in terms of tumour suppression and malignant transformation.

### FOXOs and metastasis

Numerous histopathological studies have demonstrated a connection between low FOXO expression and increased cancer metastasis [51–53]. A critical event that plays multiple roles in metastasis is the epithelial-mesenchymal transition (EMT), which is a biological process by which epithelial cells undergo changes that allow the development of a more aggressive mesenchymal cellular phenotype with the properties of stem cells [54]. EMT has, in fact, been suggested to be engaged in multiple steps of the metastasis process. A recent study shows that FOXO1 silencing using small interfering ribonucleic acid (siRNA) in hepatocellular carcinoma enhances mesenchymal and reduces epithelial marker expression, as in EMT [55]. The study also reveals that EMT induced by zinc finger E-box-binding homeobox 2 (ZEB2) can be suppressed by FOXO1 overexpression [55]. The metastasis suppressor gene nm23-H1 is involved in restricting the progression of a number of human cancers, including non-small cell lung cancer (NSCLC), and has also been shown to be positively regulated by FOXO1 in lung cancer [56]. In addition to FOXO1, FOXO3 is also strongly associated with the metastasis of multiple malignancies, including breast, pancreatic and kidney cancers [57–59]. Although overwhelming evidence has pinpointed FOXOs as suppressors of cell migration and metastasis, some literature suggests that FOXOs have an otherwise oncogenic role. Some correlation studies have linked high FOXO expression with poor cancer patient prognosis and enhanced metastasis [60–67]. Specifically, high FOXO1 and FOXO3 expression has been correlated with matrix metalloproteinase (MMP) upregulation and enhanced cancer metastasis [68, 69]. Overexpression of FOXO1 can also promote the podocyte EMT induced by high glucose conditions [70]. Equally, FOXO3 has been demonstrated in many studies to promote cell invasion and migration through inducing the expression of MMPs, including MMP-2, MMP-3, MMP-9 and MMP-13 [69, 71–73]. However, it is also notable that these studies have been centred upon normal endothelial cells. Although FOXO4 has been shown to activate the expression of MMP-9 essential for vascular smooth muscle cell migration, it also inhibits gastric cancer cell proliferation and migration [74, 75]. The fact that FOXOs are involved in regulating cell migration is further supported by the observation that FOXO6 regulates Plxna4-mediated neuronal migration during development [76]. However, when overexpressed, FOXO6 has also been shown to inhibit breast cancer cell migration and invasion [77]. These conflicting findings may reflect the cell type–specific roles of FOXOs in cell migration and metastasis. It is possible that FOXOs have a pro-metastatic function in normal endothelial cells but switch to a tumour-suppressive anti-metastatic role in cancer cells. On the whole, these findings support the hypothesis that FOXO proteins are genuine tumour suppressors but the underlying mechanisms involved in this switch from normal to cancer cells need further elucidation.

### FOXOs and angiogenesis

Angiogenesis is the process whereby new blood vessels are formed. This process facilitates the delivery of oxygen and nutrients to the target tissues and cells. These functions also make angiogenesis essential for the spreading and establishment of tumour metastases. Essentially, angiogenesis facilitates the escape of tumour cells into the bloodstream and the establishment of metastatic colonies at secondary sites. In a manner similar to angiogenesis, lymphangiogenesis also helps to disseminate lymphatic metastases. Insights from human endothelial cell studies and gene knockout mouse models have revealed a clear role for FOXO proteins in regulating the angiogenic activity of endothelial cells and blood vessel formation [23, 78]. Indeed, overexpression of constitutively active FOXO1 or FOXO3, but surprisingly not FOXO4, significantly inhibits endothelial tube formation and migration. Appropriately, FOXO1 and FOXO3 are, in fact, the most abundant FOXO proteins in mature endothelial cells. Consistent with this, silencing of either FOXO1 or FOXO3 also leads to a significant increase in the migratory and sprout-forming capacity of endothelial cells [78]. In concordance with a key role for FOXOs in angiogenesis, further gene expression profiling analysis also reveals that endogenous FOXO1 and FOXO3 negatively regulate a set of angiogenesis-related and vascular remodelling genes, including angiopoietin 2 (Ang2) and eNOS, to repress blood vessel formation and maturation [78]. The role of FOXOs in angiogenesis is further supported by studies in FOXO1/3/4 triple-knockout mice showing that FOXO depletion potentiates angiogenesis in mouse liver in response to simulation by pro-angiogenic growth factors, such as vascular endothelial growth factor (VEGF) and basic fibroblast growth factor (bFGF) [23]. Further work using the FOXO1/3/4 knockout mouse system confirms that FOXOs directly regulate the expression of endothelial cell morphogenic and vascular homeostatic mediators, including Sprouty2 and PBX1, which control the process of angiogenesis [23]. FOXO3 has also been reported to repress the expression of the potent angiogenic growth factor VEGF at the promoter level in breast cancer [31]. Similarly, FOXO1 has also been found to regulate VEGFA expression and promote angiogenesis [79].

## Deregulation of FOXOs in cancer and metastasis

### FOXO mutation in cancer

The human FOXO1 (also known as FKHR; forkhead in rhabdomyosarcoma) was first identified as the fusion partner of paired box protein (PAX) 3/7 in alveolar rhabdomyosarcoma, a paediatric tumour of skeletal muscle. The majority of highly metastatic and aggressive alveolar rhabdomyosarcomas harbour the FOXO1-PAX3/7 protein, resulting from t(2;13)(q35;q14) and t(1;13)(p36;q14) chromosomal translocation, respectively [80, 81]. Both PAX3 and PAX7 are important regulators of myogenesis [82], and their fusion proteins with FOXO1 carry the FOXO1 transactivation domain which confers a gain-of-function oncogenic PAX3/7 phenotype. These genetic changes are somatic and not inherited in the family. A G protein–coupled receptor (GPCR) Cnr1 has previously been shown to induce PAX3-FOXO1 expression to augment cell invasive capacity in alveolar rhabdomyosarcoma. From the study, the use of Cnr1 antagonist or genetic deletion attenuates migration of alveolar rhabdomyosarcoma to the lungs [83]. In a similar manner to the FOXO1 chromosomal translocation in alveolar rhabdomyosarcoma, FOXO3 (also known as FKHRL1) and FOXO4 (also known as AFX) are translocated to the MLL gene in mixed lineage leukaemia (MLL), an aggressive paediatric blood cancer [11]. MLL is characterised by the presence of a MLL-FOXO3/4 fusion protein, resulting from the t(6;11)(q21;q23) and t(X;11)(q13.1;q23) chromosomal translocation, respectively [84, 85]. Importantly, these genetic rearrangements in alveolar rhabdomyosarcoma and MLLs not only create new oncogenic fusion proteins but also cause the loss of FOXO allele from the original chromosome, resulting in FOXO locus haploinsufficiency [86]. Unlike the other FOXOs, FOXO6 has not been shown to be involved in chromosomal translocation. It has also been reported that the FOXO3 is a tumour suppressor gene commonly deleted during early-stage lung adenocarcinoma carcinogenesis [87, 88]. Characterisation of a common chromosomal 6q21 deletion in mature B cell lymphomas and childhood acute lymphoblastic leukaemia has also uncovered FOXO3 as being one of the three tumour suppressor genes frequently deleted in these rare blood cancers [89]. A loss of FOXO1 in a chromosomal deletion at 13q14 is associated with tumorigenesis of the benign mammary and vaginal myofibroblastomas [90]. Similarly, deletion of FOXO3 at 6q21 is shown to be linked with a rare highly aggressive lymphoid malignancy, natural killer cell neoplasms [91]. However, despite these examples of translocation and deletion of FOXO genes in alveolar rhabdomyosarcoma, mixed lineage leukaemia, lung adenocarcinoma and natural killer cell neoplasms, FOXO genes are generally rarely mutated in human cancers, suggesting deregulated FOXO expression and function as the predominant mechanism for cancer development and progression. The reason for the lack of FOXO mutations in cancer is unclear. However, it has been shown that a monoallelic 13q14 deletion in mammalian cells can result in a reduction in FOXO1 expression levels and cellular proliferation stress, which can, in turn, cause oxidative stress to activate the p38 MAPK to induce cellular senescence *via* the oestrogen receptor (ER) stress-activating transcription factor 6 (ATF6) axis [92]. This might represent a tumour suppressor loss–induced senescence mechanism to prevent the loss of FOXO genes [93].

### Regulation of FOXOs by post-translational modifications

**Fig. 1:**
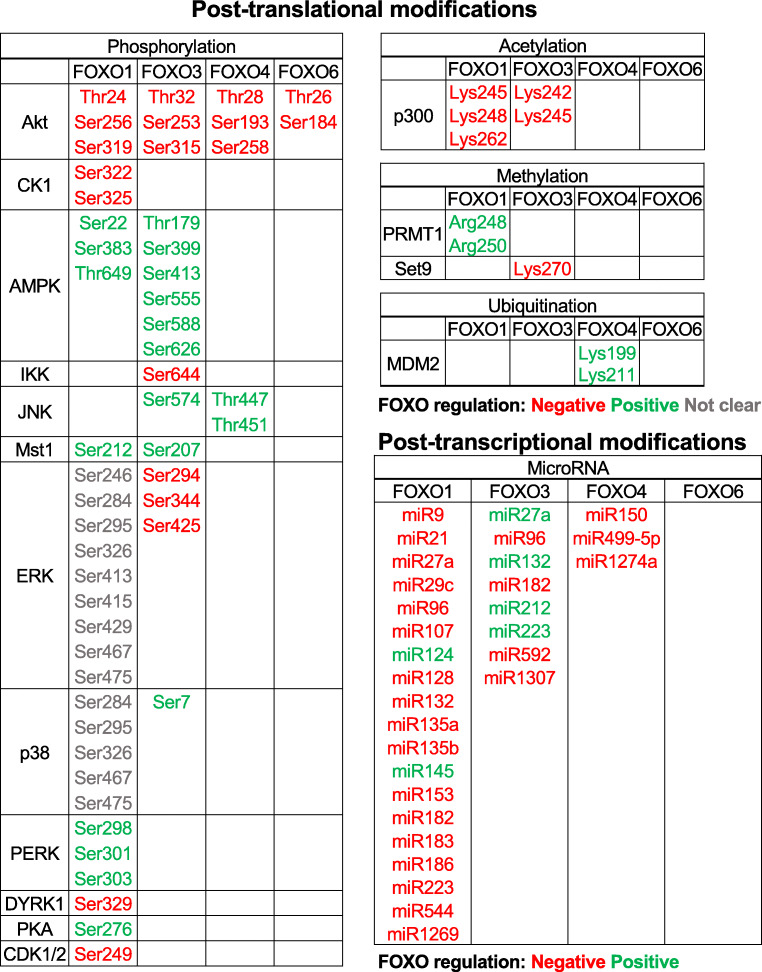
Compilation of post-translational modifications and post-transcriptional controls of FOXO proteins. At the protein level, multiple signalling pathways from different sources converge to regulate FOXO subcellular localisation, activity and stability. At the post-transcriptional level, microRNA regulates FOXO proteins to modulate cell proliferation, invasion and metastasis. Regulation: red, negative; green, positive; and grey, not known. Amino acids: threonine (Thr), lysine (Lys), serine (Ser) and arginine (Arg)

As mentioned, FOXOs are predominantly deregulated at the post-translational level in most cancers. In response to external stimuli, FOXO proteins are rapidly reversible-regulated by multiple layers of post-translational and post-transcriptional modifications including phosphorylation, acetylation, ubiquitination, glycosylation and methylation (Fig. 1). These modifications control FOXO functions through adjusting their turnover (stability), altering their subcellular localisation, changing their DNA binding affinity, controlling their transcriptional activity and regulating their interaction with co-factors. For example, these post-translational modifications can induce FOXO conformational changes and introduce new binding motifs for FOXO-binding proteins, which, in turn, regulate the expression, subcellular localisation, DNA binding and transcriptional activity of FOXOs [86]. FOXO proteins are best studied for their regulation *via* phosphorylation at different conserved serine and threonine amino acid residues predominantly by the insulin and cellular stress pathways [30].

### FOXOs and Akt-mediated phosphorylation

**Fig. 2:**
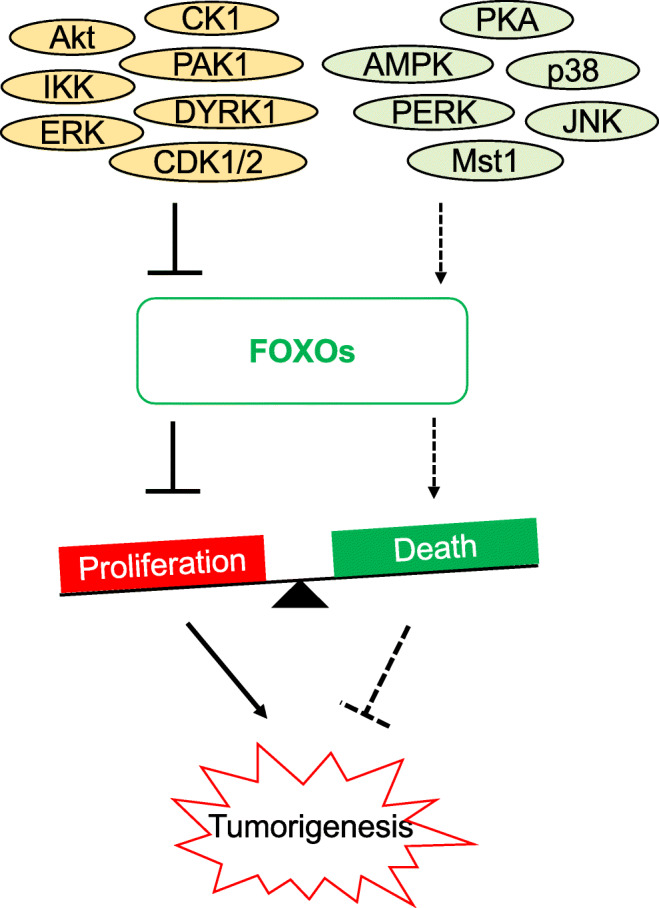
FOXO regulation by phosphorylation. Signals from various sources induce signalling pathways to regulate FOXO activity. Insulin signalling pathway induces Akt-mediated FOXO inhibition and facilitates 14-3-3 protein binding and nuclear exclusion. Subsequently, the cytoplasmic FOXO is ubiquitinated by SKP2 for protein degradation. This 14-3-3-bound FOXO can be rescued by JNK phosphorylation and PP2A dephosphorylation. Stress-activated p38, JNK and PERK promote FOXO transcriptional activity. In parallel, AMP sensor detects low intracellular energy and promotes FOXO *via* AMPK phosphorylation. However, stress-activated ERK inhibits FOXO activity by promoting MDM2-mediated polyubiquitination

The growth factor–regulated PI3K-Akt (also known as PKB) signalling cascade is one of the most frequently dysregulated pathways in cancer, resulting commonly in an upregulation of Akt and thereby an attenuation of FOXO activity [94]. Consumption of glucose-containing diets increases glucose levels in the blood serum which supplies different organs as a primary source of energy. In response to high serum glucose levels, pancreatic β-cells synthesise and secrete insulin to lower the elevated blood glucose level by promoting the cellular glucose uptake using insulin-regulated glucose transporter GLUT4 through the PI3K-Akt signalling pathway [95, 96]. Indeed, cancer cells utilise glucose for proliferation and metastasis [97]. Upon induction of the insulin receptors, the activated Akt suppresses the function and alter the subcellular localisation of FOXO proteins. Specifically, the high nutrient availability triggers insulin signalling pathway to promote Akt-mediated FOXO1 (Thr24, Ser256 and Ser319), FOXO3 (Thr32, Ser253 and Ser315), FOXO4 (Thr28, Ser193 and Ser258) and FOXO6 (Thr26 and Ser184) phosphorylations [86]. Notably, FOXO6 is only phosphorylated at two sites and it is not regulated by nucleo-cytoplasmic shuttling [98]. FOXO1 (Thr24 and Ser256) and FOXO3 (Thr32 and Ser253) phosphorylations serve as docking sites for chaperone protein 14-3-3 on the N-terminus and the DNA binding domain of the FOXO protein [11]. The binding of the 14-3-3 protein is believed to expose the FOXO nuclear export sequence to promote nuclear exclusion. Similarly, the binding of the 14-3-3 to FOXO proteins reduces the access of nuclear importing machinery to nuclear localisation sequence (NLS), suggesting that the 14-3-3 interaction prevents FOXO from nuclear translocation [99]. Another study has shown that simultaneous binding of 14-3-3 on these phosphorylated sites also disrupts and affects FOXO DNA binding capability [100]. For FOXO1, the cytoplasmic sequestration is shown to be promoted by the phosphorylation at Ser256 which introduces a negative charge to the basic nuclear localisation signal sequence [101]. In response to growth factor, phosphorylation of FOXO1 by Akt at Ser319 also primes the casein kinase 1 (CK1) to phosphorylate FOXO1 further at Ser322 and Ser325 [102]. These CK1 phosphorylation sites directly promote the interaction between FOXO1 and the nuclear export machinery Ran-CRM1 complex [102]. Conversely, the Akt-mediated phosphorylated FOXO1 and FOXO3 nuclear exclusion can be rescued by dephosphorylation mediated by protein phosphatase 2A (PP2A) (Fig. 2) [103, 104].

High levels of Akt-phosphorylated FOXO proteins are correlated with poor patient overall disease-free survival rate in many cancers [105]. At the molecular level, a study shows that the Akt signalling pathway suppresses cell migration and invasion through FOXO1 to inhibit runt-related transcription factor 2 (RUNX2) transcriptional activity in prostate cancer [106]. FOXO4 has also been identified by a genome-wide RNAi screen to be a suppressor of metastasis through antagonising PI3K/AKT signal pathway and RUNX2-dependent transcription in prostate cancer [53]. Besides RUNX2, its closed relatives RUNX1 and RUNX3 are also linked to FOXO proteins in restricting cancer survival [107, 108]. However, in these cases, RUNX1 and RUNX3 bind to and cooperate with FOXO proteins to promote cell death in breast and gastric cancers. Moreover, Akt-regulated FOXO4 has been shown to promote anti-metastatic protein ANXA8 in the bile duct cancer, cholangiocarcinoma [109]. FOXO3 can also modulate cell invasion by regulating the expression of MMP-9 in glioblastoma [110]. Through FOXO6, EGFR signalling controls SRY-related HMG-box 2 (SOX2) expression in lung cancer [111]. Recently, a SOX2 knockdown experiment shows that basal cell carcinoma migration is regulated by Akt signalling pathway [58]. In agreement, another recent study shows that hypoxia-induced breast cancer cell migration is regulated by SOX2 transcription factor [112].

### Stress signals in FOXO regulation

Unlike the nutrient and growth factor–mediated PI3K-Akt signalling cascade, FOXO activity can be enhanced by the stress-activated signalling pathway. In cancer cells, stress can promote or restrict cancer growth and progression. Reactive oxygen species (ROS) levels are often elevated in cancers by various factors, including enhanced oncogenic activity and increased metabolic function [113]. In mitochondria, ROS is a by-product of oxidative phosphorylation where a large amount of energy is generated [114]. During the process, electron transport chain complex–produced superoxide is reduced into hydrogen peroxide by Sirtuin-stimulated manganese-dependent superoxide dismutase (MnSOD, also called SOD2) [115]. This FOXO-regulated SOD2 modulates cellular antioxidant capacity. A loss of FOXO function increases intracellular ROS, pushing cancer cells to rely on Warburg effect to prevent further cellular damages [11, 116]. However, lower SOD2 expression allows cancer cells to acquire beneficial DNA damages for enhanced proliferation and to obtain drug resistance mutations [11]. Similarly, chemically induced stresses from multiple chemotherapeutic treatments have also been shown to integrate with FOXO activity [30, 117]. Cancer cells control these stresses by inducing JNK to activate FOXO-mediated oxidative stress resistance [118]. Notably, the responses of FOXOs to stress are dependent on the stress intensity in different conditions and contexts.

Oxidative stress–activated JNK can also phosphorylate the 14-3-3 protein (Ser184) to enhance FOXO activity. The phosphorylation releases FOXO proteins from 14-3-3, exposing the nuclear localisation signals to promote nuclear translocation [119]. In parallel, JNK also antagonises Akt activity by phosphorylating FOXO3 at Ser574 for activation [120]. Under oxidative stress, JNK phosphorylates FOXO4 directly at Thr447 and Thr451 to promote FOXO4 nuclear localisation [118]. Similarly, oxidative stress induces mammalian Ste20-like kinase 1 (Mst1) kinase activation in Hippo signalling pathway to promote FOXO1-mediated apoptosis [121]. During oxidative stress, Mst1 phosphorylates FOXO1 (Ser212) and FOXO3 (Ser207) directly to disrupt the binding of 14-3-3 protein to promote FOXO activity [122, 123]. Recently, FOXO protein activity has been inversely linked to epidermal growth factor receptor 2 (EGFR2, also known as HER2) overexpression. A study shows that HER2 negatively regulates FOXO1 at the transcriptional level [124]. Like the insulin signalling pathway, this HER2 regulation of FOXO activity is mediated *via* the PI3K/Akt signalling pathway [125].

**Fig. 3:**
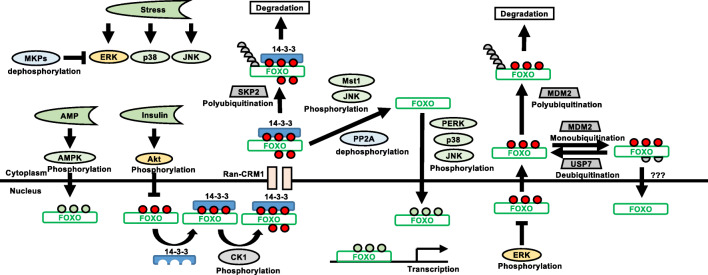
The balance of signal regulation. FOXO proteins are the point of signal integration from various sources. The intensity of these signals critically determines cell fate. Activated kinases in different pathways can either inhibit or activate FOXO transcriptional activity. A shift in signal intensity towards the inhibition of FOXO proteins allows cancer development and progression

Consistently, treatment of cancer cells with EGFR/HER2-tyrosine kinase inhibitors (EGFR/HER2-TKI; e.g. gefitinib and lapatinib) or anti-ErbB-2 monoclonal antibodies (e.g. trastuzumab) induces cell proliferative arrest and/or cell death, and this has been shown to be mediated through the PI3K/Akt/FOXO3 signalling axis [31, 126–132]. Appropriately, an HER2 upregulation induces both Akt and JNK to promote gastric cancer growth and metastasis [133]. These findings suggest that multiple stress signals converge and integrate to regulate FOXO activity and that FOXO proteins are important factors in a pivot balance to determine cell fates (Fig. 3). It is also plausible that an intermediate degree of stress promotes cell metastasis to evade from the source of stress whilst a high level of stress induces cell death.

Beside the stress-induced JNK, FOXO is also regulated by two other major MAPKs: extracellular signal–regulated kinases (ERKs) and the p38 family of stress-activated MAPKs [134]. These signalling pathways (JNK, ERK and p38) modulate the FOXO-regulated intracellular ROS cooperatively [135]. In fact, the outcome of the FOXO activity regulation is predominantly controlled by the duration and magnitude of the multiple MAPK signal integration [136]. Each of the MAPKs (JNK, ERK and p38) is regulated by mitogen-activated protein kinase phosphatases (MKPs) to control its activity and subcellular localisation, increasing the complexity of FOXO regulation [136]. Indeed, increased MKP expression elevates cancer progression and drug resistance, linking MKPs to FOXO function [137]. Although there is no evidence of a direct link between MKP and FOXO activities in human cancer, a study has demonstrated that insulin-induced MKP3 (also known as dual-specificity phosphatase 6; DUSP7) interacts and dephosphorylates FOXO1 on Ser256 in a mouse model to promote its nuclear import for gluconeogenic gene transcription [138]. Noteworthy, gluconeogenetic enzymes play a crucial part in the regulation of cancer EMT [139]. A study also shows that ERK directly phosphorylates FOXO1 at Ser246, Ser284, Ser295, Ser326, Ser413, Ser415, Ser429, Ser467 and Ser475 to alter the transcriptional activity of Ets1, a regulator of angiogenesis-related genes [134]. However, the functional consequence of the ERK/FOXO1-regulated Ets1 on the induction of angiogenesis and metastasis has yet to be confirmed in cancer cells.

In cancer, the ERK and p38 MAPK pathways cooperate to control anoikis, a form of cell death triggered by a loss of extracellular matrix (ECM) attachment [140]. Anoikis resistance, the insensitivity to anoikis-induced cell death, is a prerequisite for EMT and cancer metastasis [141]. Indeed, anoikis has been shown to be regulated by FOXO3 activity during breast cancer metastasis [142]. It has also been shown that both ERK and p38 MAPK can activate FOXO1 by phosphorylation [134]. Nine serine residues in FOXO1 are phosphorylated by ERK, whilst five of them (i.e. Ser284, Ser295, Ser326, Ser467 and Ser475) are also phosphorylated by p38 MAPK [134]. Whilst the physiological consequence of the ERK and p38 MAPK-dependent FOXO1 phosphorylation is unclear, p38 MAPK induction promotes fibroblast cell invasion and anchorage-independent cell growth [143]. In agreement, inhibition of p38 MAPK reduces glioma invasiveness [144]. MKK4 also mediates suppression of metastasis in ovarian cancer *via* activation of p38 MAPK [145]. Moreover, the upstream regulator of ERK MEK1/2 can directly induce Akt for FOXO1 phosphorylation to promote cell migration [146]. Another study shows that inhibition of both Akt and ERK signalling pathways synergistically induces FOXO transcriptional activity to inhibit angiogenesis [147]. Moreover, UV irradiation induces stress-mediated JNK to suppress both ERK and Akt activities and promotes FOXO3 nuclear translocation, probably for DNA damage repair [148]. Conversely, ERK2 can also promote cell migration and invasion through the Rac1/Cdc42 guanine nucleotide exchange factor (GEF) DOCK10, which induces JNK to activate FOXO1 and promote EMT [149]. Paradoxically, the loss of FOXO1 which is followed by an upregulation of p38 initiates a compensatory mechanism to promote other FOXO protein activities. p38 MAPK has been shown to phosphorylate FOXO3 on Ser7 to mediate nuclear localisation and senescence in response to doxorubicin-induced DNA damages in breast cancer [150]. In a similar manner, the p38 MAPK is also activated by cetuximab to induce cell death and inhibit cell proliferation *via* FOXO3 in colorectal cancer [151]. During ER stress, unfolded protein response (UPR) activates inositol-requiring enzyme 1 (IRE1), protein kinase R (PKR)-like ER kinase (PERK) and ATF6 to promote ER folding capacity and maintain cellular proteostasis for cell survival [152, 153]. Furthermore, PERK has previously been shown to promote FOXO1 activity by direct phosphorylation at Ser298, Ser301 and Ser303, representing another layer of FOXO regulation in cancer [154]. Nevertheless, a recent study has shown that PERK, instead of promoting FOXO activity, can inhibit FOXO activity through enhancing Akt activity [155]. Together, these findings suggest that cancer cell metastasis is regulated by FOXO proteins, whose activity is, in turn, modulated by stress-activated signals, including the JNK, ERK and p38 MAPKs.

### FOXO ubiquitination

The Akt-phosphorylated FOXOs (e.g. FOXO1 at Ser256) are recognised by the activated F-box protein S-phase kinase-associated protein 2 (SKP2) in the SCF^SKP2^ E3 ligase complex, which targets FOXO proteins for polyubiquitination and degradation [156]. Interestingly, FOXO3 has also been shown to be a negative regulator of SKP2 expression, and this could represent a positive feed-forward loop in the regulation of FOXO expression and activity [157]. Collectively, the Akt-phosphorylated FOXOs are sequestered in the cytoplasm, transcriptionally inactive and targeted by the ubiquitin-proteasome system for degradation. At the molecular level, ERK phosphorylates FOXO1 and FOXO3 (Ser294, Ser344 and Ser425) to recruit the MDM2 E3 ubiquitin ligase for FOXO poly-ubiquitination and proteasomal degradation [158, 159]. Besides poly-ubiquitination, a study suggests that FOXO4 can be mono-ubiquitinated at Lys199 and Lys211 by MDM2 in response to oxidative stress for nuclear translocation. The mono-ubiquitinated FOXO4 can be reversed by ubiquitin-specific protease 7 (USP7; also known as HAUSP)–mediated de-ubiquitination [160, 161]. However, the mono-ubiquitination on other FOXO proteins has not been demonstrated and the exact mechanism of this modification is still unclear (Fig. 2). As glucose is a source of energy, Akt-mediated FOXO1 ubiquitination is also interconnected with an energy homeostasis regulator, the 5′ adenosine monophosphate–activated protein kinase (AMPK). AMPK is an intracellular energy sensor which senses the ADP/ATP ratio and regulates cell growth, autophagy and metabolism [162]. A study shows that AMPK-mediated FOXO1 phosphorylation at Ser383 and Thr649 promotes FOXO protein stability, nuclear translocation and transcriptional activity [163]. A recent study also demonstrates that AMPK can phosphorylate FOXO1 at Ser22 to activate FOXO1 by blocking Akt-mediated FOXO sequestration by the 14-3-3 protein in the cytoplasm [164]. Indeed, the hypoxia-induced AMPK has been shown to promote FOXO1 activity [165]. Likewise, FOXO3 is also phosphorylated by AMPK at Thr179, Ser399, Ser413, Ser555, Ser588 and Ser626 for transcriptional activation, but this AMPK-mediated phosphorylation enhances the activity of FOXO3, without altering its subcellular localisation and DNA binding affinity [166]. Indeed, AMPK-induced FOXO3 phosphorylation has been shown to suppress pancreatic cancer growth and metastasis [167]. Of note, in response to growth factor stimulation, IκB kinase (IKK) also exclusively phosphorylates FOXO3 (Ser644) for ubiquitination and degradation as the other FOXO proteins do not have similar amino acid residues [168].

### FOXO regulation by other kinases and factors

Besides the PI3K-Akt and stress-activated MAPK signalling pathways, many other kinases and dependent cascades also contribute to FOXO regulation. For instance, the p21-activated kinase (PAK1, a downstream target of PI3K) can bind and phosphorylate FOXO1 directly to prevent its nuclear translocation in breast cancer [169]. Similarly, the transforming growth factor β–activated kinase (TAK1)-Nemo-like kinase (NLK) pathway also mediates FOXO1 phosphorylation in the transactivation domain to drive its nuclear exclusion [170]. The dual-specificity tyrosine-phosphorylated and regulated kinase 1 (DYRK1) phosphorylates FOXO1 (Ser329) to promote its activity in unstimulated quiescent mammalian cells [171, 172]. Recently, a study shows that the glucagon-activated PKA also phosphorylates FOXO1 at Ser276 to promote its nuclear relocalisation and stability in liver cancer and limit insulin-induced FOXO nuclear export and their ubiquitination-degradation [173].

During normal cell divisions, CDK/cyclin complexes are the main regulators of the cell cycle checkpoints which can influence cell fates. However, in cancer, these CDK/cyclin complexes are commonly deregulated, resulting in deregulated and highly proliferative cells [174]. Indeed, CDK1 (also called CDC2) and CDK2 have shown to phosphorylate FOXO1 at Ser249 to alter its subcellular localisation. Paradoxically, FOXO1 has also been found to be activated by CDK1 to induce apoptosis gene activation in post-mitotic neurons but FOXO1 phosphorylation by CDK1 promotes its inhibition in human prostate adenocarcinoma cells [175, 176]. These contrasting findings may reflect the deregulation of normal FOXO1 control and function in cancer cells. On the other hand, CDK2 has been confirmed to inhibit FOXO1 by promoting its nuclear exclusion to suppress FOXO1 transcriptional activity [177]. With respect to cell migration, an activator of CDK1 and CDK2, Cdc25A phosphatase can enhance FOXO1 stability to promote MMP-1-mediated metastasis in breast cancer [68]. Consistent with this finding, the FOXO1-derived small peptide FO1-6nl inhibits CDK1/2-mediated FOXO1 phosphorylation and prevents prostate cancer proliferation [178]. In addition, FOXO3 circular RNA (circ-FOXO3) has been reported to form an inhibitory complex with CDK2 and p21^Cip1^ to restrict cell cycle progression [179]. During cell division, genotoxic stress, induced by cytotoxic chemotherapeutic agents, can revert the FOXO phosphorylation state and induce a senescent and/or apoptotic response in cancer cells [64, 126, 127, 129, 151, 155, 177, 180–184]. On the other hand, FOXO3 can also upregulate the expression of DNA damage–inducible 45 (GADD45) of the DNA repair mechanism and has a role in ATM-mediated DNA damage response to prevent cancer from acquiring further mutations [185, 186]. These findings further emphasise the importance of FOXO3 in the prevention of cancer propagation and progression.

### FOXO acetylation

Although mammalian cells have protein kinases in place to regulate FOXO activity, another post-translational modification (PTM) acetylation applies extra layers of control to fine-tune their cellular functions. In general, depending on the sites of modification, acetylation of nuclear proteins usually alters their DNA binding affinity and, hence, their activity [187]. For FOXO proteins, acetylation occurs predominantly at the Wing2 region of the forkhead DNA binding domain, where FOXO proteins were used for recognising their DNA consensus sequences on target genes [188]; however, the functional consequence of FOXO acetylation is not clear-cut. An earlier report suggests that acetylation of sites Lys242, Lys245 and Lys262 located within the DNA binding domain of FOXO1 attenuates its transcriptional activity [189]. In support of this, it has been demonstrated that oxidative stress–induced CREB-binding protein (CBP)/p300 acetylates FOXO1 at Lys245, Lys248 and Lys262 to restrict its DNA binding in cancer [190]. However, another subsequent crystallographic structural analysis suggests that acetylation at equivalent sites (Lys242 and Lys245) in FOXO3 enhances its DNA binding and, therefore, its transcriptional activity [191]. For FOXO1, there is also a controversial link between phosphorylation and acetylation. One study shows that FOXO1 acetylation requires insulin-induced Akt phosphorylation to prime the modification [192]. Yet, another suggests that p300-mediated acetylation can increase sensitivity for the inhibitory FOXO1 phosphorylation at Ser253 [189]. Despite the controversy over the exact order in which these modifications occur, these data suggest that acetylation and Akt-mediated phosphorylation cooperate to inhibit FOXO1 activity. Upon acetylation, FOXO1 has also been shown to have a reduced affinity for the genomic compacting protein nucleosomes, affecting multiple gene accessibility for other transcription factors [193].

FOXO acetylation is primarily mediated by CBP/p300 and reversed by Sirtuins (SIRTs), which are a group of highly conserved NAD-dependent deacetylases which have many roles in epigenetics and human diseases, including cancer [194]. An earlier study suggests that SIRT2-mediated deacetylation promotes FOXO3 transactivation activity to reduce cellular ROS and induce cell death in cancer cells [195]. However, a later study by the same research group has shown that SIRT1 and SIRT2 mediate deacetylation of FOXO3 to promote FOXO3 ubiquitination and degradation in Skp2-dependent manner, implying that acetylation increases FOXO stability and enhances its tumour-suppressive function [196]. The discrepancy between the two studies is likely due to the overlapping and compensatory roles played by SIRT1 and SIRT2 in cancer, whilst inhibition of one leads to the induction of the other [197]. In support of a tumour-suppressive role for FOXO acetylation, a report shows that the acetylation recruits FOXO1 to promyelocytic leukaemia (PML) protein in the nucleus to protect FOXO1 against ubiquitination and to promote its activity in pancreatic beta cells [198]. In agreement, the interaction between FOXO3 and PML has also been observed in breast cancer [199]. In addition, another study shows that four-and-a-half LIM 2 (FHL2) interacts with FOXO1 to facilitate SIRT1-mediated deacetylation to reduce FOXO1 activity in prostate cancer [188]. SIRT6 also represses FOXO3 acetylation to promote clonal renewal and survival in breast cancer [200]. Similarly, FOXO4 acetylation induces apoptosis of podocytes in diabetes through activating the pro-apoptotic gene Bim (Bcl-2l11) [201]. Studies on mitochondrial reprogramming have produced further evidence that SIRTs repress FOXO acetylation to promote cancer progression and metastasis. Elevated ROS in cancer has been shown to lead to mitochondrial reprogramming *via* the evolutionarily conserved SIRT-FOXO-SOD2 axis to neutralise oxidative stress and promote cancer survival and metastasis [202, 203]. Besides SIRTs, the histone deacetylase HDAC3 has been shown to be specifically recruited by geminin to FOXO3 to facilitate FOXO3 deacetylation and breast cancer metastasis [57]. In spite of this tumour-suppressive protective role, geminin is also frequently overexpressed in human cancers. Collectively, the evidence accumulated to date suggests that FOXO acetylation is mediated by EP300/CBP and is reversed by SIRTs in general, promoting the tumour-suppressive function of FOXOs by increasing the transcription of genes important for cell proliferative arrest, senescence and cell death.

Besides its tumour-suppressive function, recent evidence also reveals that FOXO acetylation is also targeted by anticancer chemotherapeutics to mediate their cytotoxic and cytostatic function. For example, it has been reported that capsaicin treatment induces CBP and represses SIRT1 expression, resulting in an increase in FOXO1 acetylation to limit pancreatic tumour growth [204]. Moreover, SIRT6 can promote paclitaxel and epirubicin resistance in breast cancer [200]. EP300 and SIRT1/6 also co-regulate the cytotoxic function of the EGFR/HER2 inhibitor lapatinib through modulating FOXO3 acetylation and activity in breast cancer [129]. Similarly, SIRT2-mediated FOXO3 deacetylation has also been implicated in lapatinib response and sensitivity, and that SIRT2 can specifically antagonise the cytotoxicity of lapatinib through mediating FOXO3 deacetylation in both sensitive and resistant nasopharyngeal carcinoma (NPC) cells. The synthetic glucocorticoid dexamethasone also targets FOXO3 phosphorylation on Ser7 and acetylation on Lys242/Lys245 to mediate its cytotoxic function in B acute lymphoblastic leukaemia (B-ALL) [180]. These findings also propose that SIRTs can be important biomarkers for metastatic and drug-resistant clones and that targeting the SIRT-FOXO3 axis may provide novel strategies for treating cancer and for overcoming chemoresistance. Indeed, specific as well as pan-SIRT inhibitors have proved to be effective in tackling cancer and in overcoming cancer drug resistance [129, 197, 205, 206].

### Other FOXO PTMs

Methylation is a post-translational modification which introduces a methyl group to proteins. Protein methylation is predominantly associated with histone methylation which modulates gene expression [207]. However, FOXO proteins are also direct targets of methylation. For instance, the ubiquitously expressed and tissue-specific protein arginine *N*-methyltransferase 1 (PRMT1) has been reported to methylate FOXO1 at Arg248 and Arg250 to block the Akt-mediated Ser253 phosphorylation and inhibition [208]. The lysine methyltransferase Set9 has also been demonstrated to methylate FOXO3. This Set9-mediated FOXO3 methylation at Lys270 leads to attenuation of its DNA binding activity and downregulation of transactivation without affecting the Akt-mediated phosphorylation, its protein stability and subcellular localisation [209]. On the evidence of these studies, FOXO methylation appears to promote the activity of FOXO proteins. In fact, protein methylation has been shown to have a role in protein-protein interaction, DNA binding affinity, protein stability and subcellular localisation [210].

It is also unclear whether like methylation, other less common post-translational modifications (i.e. glutathionylation, glycosylation, SUMOylation, hydroxylation, neddylation, citrullination, prenylation, palmitoylation, myristoylation and *s*-nitrosylation) also directly modify FOXO proteins and if they are involved in modulating FOXO activity in cancer [211]. For example, neddylation inactivation by the specific inhibitor MLN4924 can prevent FOXO3 nuclear export, decrease its binding to the ESR1 (oestrogen receptor) gene promoter and improve fulvestrant sensitivity in breast cancer. Nevertheless, in this case, it is still unknown whether FOXO3 is directly modified by neddylation and if FOXO3 neddylation has a role in modulating its activity [212].

### Protein-protein interactions in FOXO regulation

Apart from the post-translational modifications, protein-protein interactions also modulate FOXO function and activity. Peroxisome proliferator–activated receptors (PPARs) are a family of ligand-activated nuclear receptor transcription factors that function downstream of ERK to regulate cell metabolism and adipocyte differentiation [213]. In this respect, FOXO1 and PPAR can compete for ERK signals and thereby antagonise the transcriptional activity of one another [214]. In liver carcinoma cells, PPAR can also interfere with the binding of apolipoprotein C III (ApoC-III) to FOXO1, affecting the lipid metabolism and serum triglyceride levels [215, 216]. ApoC-III plays a key role in the regulation of triglyceride metabolism and has been suggested to be a predictive marker for NSCLC [217]. Nonetheless, lipid metabolism and lipid-mediated signalling are a key to cancer metastasis [218], and dysregulated lipid metabolic enzymes are known to be associated with cancer cell invasion and metastasis [219].

In the nucleus, androgen receptor (AR) has been reported to interact with FOXO1. The nuclear receptor AR, which is expressed in a vast range of tissue, responds to the male sex hormone androgen for activation [220]. Indeed, the development of prostate cancer is intimately associated with androgen. In prostate cancer, elevated AR expression promotes EMT [221]. Likewise, AR promotes haematogenous metastasis and angiogenesis of clear cell renal cell carcinoma (CCRCC) [222]. In prostate cancer, an activated nuclear AR binds to and reduces the DNA binding capacity of FOXO1 in an Akt-independent manner, affecting the expression of the proapoptotic genes, such as Fas [223]. Thus, AR binding generally reduces the tumour-suppressive function of FOXO proteins. ER is another hormone-regulated receptor that has a strong link with cancer metastasis. ER is overexpressed in multiple cancers, including breast and ovarian cancers [224]. In breast cancer, ER, encoded by oestrogen receptor alpha gene (ESR1), is commonly deregulated by gene amplification, point mutations and genetic fusion, leading to enhanced cancer invasion and metastasis [225]. The oestrogen-activated ER has been shown to interact with FOXO1 to repress its activity, and it is speculated that this interaction may play a part in the regulation of cancer metastasis [226].

Poly(ADP-ribose) polymerase 1 (PARP1) is a nuclear protein important for the regulation of DNA damage repair, chromatin remodelling, cell death and gene transcription [227, 228]. PARP1 has been shown to promote cell metastasis in melanoma and in prostate and lung cancers [229–231]. FOXO1 interacts with PARP1and gets poly(ADP-ribosyl)ated in an Akt-independent manner, resulting in suppression of FOXO1 transcriptional activity and cell proliferation [232]. In cancer cells, PPARγ co-activator 1α (PGC-1α) interacts with FOXO1 and acts as a co-activator in hepatocytes for activation of gluconeogenic genes. The study shows that the interaction is involved in the cellular oxidative stress protection and can be interrupted by insulin-mediated Akt phosphorylation [233, 234]. Moreover, FOXO1 has been shown to regulate PGC-1α at the transcript level, suggesting a feedback mechanism for oxidative stress regulation [235]. Like PGC-1α, FOXO1 also interacts with C/EBPα to promote the expression of gluconeogenic genes, including phosphoenolpyruvate carboxykinase (PEPCK) [236]. The gluconeogenic enzymes are known to affect cancer growth [237, 238]. Recently, gluconeogenesis has also been shown to be upregulated in brain metastatic breast cancer cells, suggesting that an increase in energy production can promote cancer metastasis *via* FOXOs [239].

Fanconi anaemia, complementation group D2 (FANCD2) is an important DNA damage response protein, which is activated during DNA replication as well as DNA damage repair [240]. In the rare genetic disease Fanconi anaemia (FA), the loss of FANCD2 activity has been shown to contribute to an accumulation of genetic mutations and therefore promote leukaemogenesis or tumorigenesis [240]. In cancer cells, FANCD2 also interacts with FOXO3 to regulate antioxidant gene expression in response to oxidative stress [241]. Whilst the interaction between FANCD2 and FOXO3 has not been directly linked to cancer metastasis, FANCD2 overexpression on its own has been shown to be correlated with lymph node metastasis of colon cancer [242]. Elevated FANCD2 expression also increases cancer therapy tolerance and is associated with an increased risk of metastasis [243]. Indeed, induction of antioxidant gene expression by FANCD2 and FOXO3 can also impact on cancer therapy resistance as radiotherapy and chemotherapeutic drugs can influence cancer treatment outcome through their modulation of ROS [244, 245]. Notably, in parallel to FOXO protein regulation, MYC, which controls at least 15% of the entire genome, is also regulated by the PI3K-Akt pathway [246]. With similar upstream regulation, the oncogenic MYC also competes with FOXO3 for the binding of promoter regions of genes, including p27^Kip1^ [247]. The p27^Kip1^ is a CDK inhibitor which regulates cell cycle progression, and it is often downregulated in cancer [248]. FOXO proteins also cooperate with other FOX proteins to bind to and drive the transcription of target genes. For example, FOXO1 and FOXA1/A2 have been shown to facilitate one another’s binding and collaborate to open chromatin at insulin-regulated genes [20, 21]. Furthermore, FOXO1 also cooperates with FOXA1 to function as pioneer factors to facilitate AR binding in prostate cancer [249]. In other words, FOXOs also work together with other FOX proteins to facilitate the recruitment of other transcriptional elements to promote the transcription of target genes.

### FOXO post-transcriptional regulation by microRNAs

MicroRNAs (miRs) are small non-coding RNAs (about 22 nucleotides) that participate in RNA silencing, and these small RNAs also participate in the regulation of FOXO expression post-transcriptionally through inducing FOXO messenger RNA (mRNA) degradation, translation inhibition and gene silencing (Fig. 1) [250, 251]. MicroRNAs, such as miR-107, miR-132, miR-223 and miR-1269, have been shown to negatively regulate FOXO1 expression to promote cell proliferation in a number of cancer cells [252–255]. Likewise, miR-21 promotes the proliferation of pancreatic ductal adenocarcinoma (PDAC) through repressing FOXO1 expression [256, 257]. Notably, the miR-21 has also been shown to regulate cancer metastasis [251]. Interestingly, miR-21 can also promote growth, metastasis and drug resistance in cancer cells by targeting phosphatase and tensin (PTEN) homologue, an upstream repressor of Akt-PI3K activity and, therefore, an indirect activator of FOXOs [258]. Thus, miRs, like miR-21, regulate not only FOXO expression but also their activity at the same time [259]. Wnt/beta-catenin activates miR-183/miR-96/miR-182 expression in hepatocellular carcinoma to promote cancer cell invasion. Meanwhile, a similar group of microRNAs, miR-193, miR-96 and miR-182, also suppress FOXO1 expression in hepatocellular carcinoma, resulting in enhanced cell invasion and metastasis [260]. A similar outcome is also observed with FOXO1, when targeted by miR-135a and miR-544 [261, 262]. In endometrial cancer, miR-9, miR-27a, miR-96, miR-128, miR-153, miR-183 and miR-186 also function cooperatively to suppress FOXO1-regulated cell cycle arrest and cell death [263]. The concerted effort of this group of miRNAs to suppress FOXO1 renders depletion of one miR species having no effects on FOXO1 expression or endometrial cancer cell proliferation and survival [263]. The microRNA miR-29c also represses FOXO1 expression to facilitate breast cancer cell growth, migration and invasion [264], whilst miR-135b inhibits FOXO1 expression to promote cell proliferation and invasion in osteosarcoma [265]. In contrast, miR-124 and miR-145 can enhance FOXO1 activity to promote cell cycle arrest and delay cell proliferation, respectively [266, 267].

Interestingly, miR-96 is found to promote colorectal cancer proliferation through targeting both FOXO1 and FOXO3, suggesting that there are common complementary sequences in FOXOs for miR binding [268]. Similarly, miR-182 suppresses both FOXO1 and FOXO3 expression levels in prostate and skin cancer, respectively, to promote cancer cell migration and invasion [269, 270]. In agreement, a study with anti-miR-182 mimetics in the xenograft mouse model has shown that miR-182 inhibition can restrict ovarian cancer cell invasion and metastasis *in vivo* [271]. Furthermore, miR-132, miR-223 and miR-27a can induce proliferative arrest and cancer cell death through FOXO1 and FOXO3 [272]. In Alzheimer disease, miR-132 and miR-212 also control FOXO3-mediated neuronal apoptosis [273]. FOXO3 is also targeted by miR-592 and miR-1307 to promote colorectal cancer metastasis and prostate cancer proliferation, respectively [274, 275]. FOXO4 is targeted by miR-499-5p and miR-1274a for enhanced cell metastasis in colon and gastric cancers, respectively [276, 277]. Similarly, miR-150 restricts the expression of FOXO4 to promote cervical cancer proliferation and lung cancer cell metastasis [278, 279]. Not only FOXO expression and activity are regulated by miRs; evidence also exists that miRs are also regulated by FOXO proteins. In fact, cross-talks between miR-155-5p and FOXO3 have been shown to modulate cell growth in lung cancer [280]. The miR-155 has also been associated with circular RNA FOXO3 (circ-FOXO3) and FOXO3 in lung cancer migration and invasion [281]. The circ-FOXO3 shares identical sequences with FOXO3 mRNA and can buffer several miRs from interacting with FOXO3 mRNA. Thus, circ-FOXO3 can upregulate FOXO3 expression by preventing the binding of miR to the FOXO3 mRNA [282]. Furthermore, FOXO3 upregulates miR-622 to suppress hypoxia-inducible factor 1 alpha (HIF-1α) to induce cell invasion and migration in lung cancer [283]. FOXO3 has also been shown to bind and activate miR-34 for β-catenin inhibition which subsequently suppresses cell migration in prostate cancer [284]. Whilst many reports have documented the direct relationships between miR and FOXO expression levels, another microRNA (miR-205) has been shown to antagonise Akt signalling in lung cancer, providing an indirect route to activate FOXO activity [285]. Although miRs have been established to play a key part in regulating FOXO expression and cancer metastasis, how these miRs are regulated themselves remains to be explored. Nonetheless, these findings suggest that post-transcriptional regulation of FOXO expression is important for the regulation of FOXO activity in the modulation tumorigenesis and metastasis.

### Cross-communication between FOXOs and other FOX proteins

**Fig. 4:**
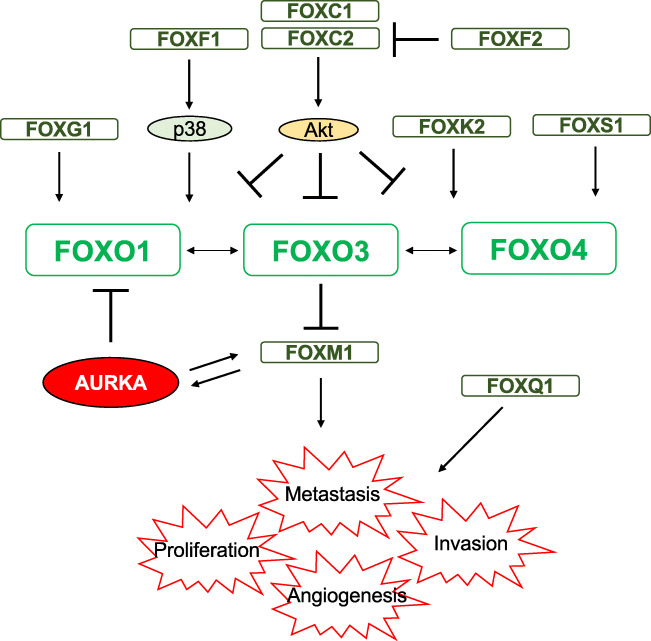
Relationships between FOX proteins. Apart from the conventional signalling pathways, FOXO proteins are also regulated by other FOX proteins which modulate FOXO activity. FOXO proteins are the central axis of many signalling pathways which determine cell fates. Downregulation of FOXO proteins enhances cell proliferation, invasion, metastasis and angiogenesis

In mammals, there are 50 known FOX proteins. Together, they regulate a wide range of biological processes encompassing cell proliferation, survival, differentiation, migration and stress response. These FOX proteins share highly homologous DNA binding domains and recognise overlapping gene targets. In consequence, tissue-specific misregulation or misexpression of these FOX genes can lead to a gain of function and result in cancer initiation and progression. Recent evidence also suggests these FOX proteins share several attributes, and intriguingly, one of them common to the majority of FOX proteins is their regulation of cancer metastasis. Apart from having overlapping functions, the expression and activity of FOXOs are also influenced directly and indirectly by their close cousins, such as FOXM1, FOXC1, FOXC2, FOXF1, FOXS1, FOXG1 and FOXK2 (Fig. 4) [286]. FOXM1 (also known as HFH-11B, Trident, Win and MPP2) is an oncogenic transcription factor which is known to be overexpressed during early cancer development [287]. It is also a potent regulator of metastasis [288]. To date, many studies have documented that FOXM1 is a key regulator of cancer cell proliferation, DNA damage repair, cancer drug resistance, metastasis and invasion [289, 290]. The tumour suppressor FOXO3 can antagonise the oncogenic activity of FOXM1 in a number of ways. Apart from FOXO3 and FOXM1 having opposite effects on chromatin remodelling of common target genes, FOXO3 also competes with FOXM1 for DNA binding at target genes and directly represses FOXM1 expression at the promoter level [2]. These multiple levels of control of FOXM1 by FOXO3 emphasise the significance of FOXO3 as a tumour suppressor. FOXO3 and FOXM1 are also intimately associated with chemotherapeutic drug resistance and cancer stem cell properties [30, 289–291]. Furthermore, the FOXO3-FOXM1 axis has been shown to regulate VEGF, an important factor for angiogenesis and metastasis [31]. Indeed, targeting FOXM1 has been shown to significantly inhibit cancer growth and metastasis [292]. Interestingly, FOXM1 and Aurora kinase A (AURKA) promote the activity and expression of each other in a positive feedback loop [293]. AURKA has also been shown to negatively regulate FOXO1 at the transcriptional level, indicating that FOXM1 can indirectly regulate FOXO expression *via* AURKA to sustain its expression and activity in cancer cells [294]. Likewise, Akt and FOXM1, both upstream inhibitors of FOXO3, also positively regulate cell migration in a positive feedback loop manner to promote tongue squamous cell carcinoma cell migration [295]. FOXC1 (also known as FKHL7) is another forkhead protein closely related to metastasis and FOXO function. High FOXC1 expression has been found to be associated with highly metastatic colon, breast and liver cancers [296–299]. FOXC1 has also been shown to induce MMP-7 expression to promote breast cancer metastasis [299]. Furthermore, overexpression of FOXC1 promotes tumour metastasis and predicts poor prognosis in liver cancer [296]. Conversely, knockdown of FOXC1 reduces the expression of mesenchymal genes, such as vimentin, fibronectin and N-cadherin [300]. Furthermore, FOXC1 knockdown displays lower expression levels of MMP-1, MMP-2, MMP-7 and MMP-9 with reduced mesenchymal characteristics and metastatic capability [301]. In melanoma, FOXC1 has been shown to promote tumorigenesis by activating the MST1R/PI3K/Akt pathway and is associated with poor prognosis [302]. Thus, FOXC1 can repress FOXO activity through enhancing the PI3K/Akt signalling. Similarly, FOXC2 (also known as MGH1 and FKHL14) has been linked to metastasis in breast cancer, colorectal cancer (CRC) and osteosarcoma [303–306]. FOXC2 has been demonstrated to also upregulate ERK and Akt activities to mediate FOXO3 inhibition [307]. Anoikis can also induce FOXC2 expression to facilitate cancer migration in osteosarcoma [303]. This anoikis-induced osteosarcoma cell invasiveness and metastasis to the lung are dependent on the ability of FOXC2 to induce CXCR4, a chemokine receptor responsible for tumour growth, invasion, angiogenesis, metastasis, relapse and therapeutic resistance [308]. High FOXC2 expression is strongly correlated with invasion and metastasis of CRC [304], whereas downregulation of FOXC2 reduces colon cancer invasiveness and their metastatic potential [304]. In concordance, FOXC2 has been found to be required for EMT and the display of mesenchymal-like properties in breast cancer [305, 306]. FOXF1 (also known as FKHL5) is essential for mesenchymal cell migration [309]. Previously, FOXF1 has been shown to induce p38 MAPK activity, one of the upstream activators of FOXO1 and FOXO3 [310]. Similarly, FOXF1 suppresses osteosarcoma cell invasion and migration by decreasing MMP-2 and MMP-9 expression levels [311]. In agreement, FOXF1 can be promoted by the tumour suppressor p53 to induce the expression of E-cadherin (the epithelial cadherin), an epithelial cell marker [312]. Although FOXF1 has not been directly associated with FOXOs, it is plausible that FOXF1 can induce p38 MAPK to activate FOXO activity. On the contrary, FOXF1 has also been found to induce EMT and angiogenesis by activating SNAI1 and VEGFA, respectively, to promote cancer metastasis and angiogenesis in colon cancer [313, 314]. In the tumour microenvironment, FOXF1 also stimulates cancer-associated fibroblasts to facilitate lung cancer tumorigenesis and migration [315]. Based on this evidence, it is likely that FOXF1 has different metastatic and angiogenic roles in epithelial and mesenchymal cells. FOXS1 (also known as FKHL18) has been shown to suppress the transcriptional activity of FOXO3 and FOXO4 [316]. Although FOXS1 is predominately expressed in the nervous system, overexpression of FOXS1 can inhibit gastric cancer proliferation and metastasis [317]. Microarray analysis of metastasis-associated mRNA also shows that FOXS1 is downregulated in high metastatic lung cancer [318]. The forkhead protein FOXG1 (previously known as BF1) forms a complex with FOXO and SMAD proteins to promote neuronal differentiation [319]. Notably, SMAD3 and SMAD4 also interact with FOXO1 to promote the transcription of p21Cip1, an important negative cell cycle regulator [320]. Whilst the FOXG1-FOXO1 complex has not been proved to be directly linked to cell metastasis, suppression of FOXG1 by miR-200b can promote cell proliferation and metastasis in cervical cancer [321]. Furthermore, miR-422a also negatively regulates FOXG1 expression to modulate liver cancer metastasis [322]. FOXK2 has also been found to regulate FOXO3 expression at the transcriptional level to mediate the cytotoxic effects of epirubicin and paclitaxel in breast cancer [323]. Interestingly, like FOXO3, FOXK2 has been shown to be able to repress breast cancer carcinogenesis [324].

Although a direct relationship between FOXOs and some of the other FOX proteins has yet to be established, these FOX proteins have already been shown to have a key role in cancer cell metastasis. For instance, FOXQ1 has been shown to modulate EMT in breast, gastric, bladder and cervical cancers [325–328]. FOXQ1 also promotes metastasis by transactivating ZEB2 and VersicanV1 expression in liver cancer [329]. Likewise, FOXQ1 also negatively regulates CDH1, which encodes epithelial cadherin or E-cadherin, to promote oesophageal squamous cell carcinoma metastasis [330]. In colon cancer, FOXQ1 transactivates Twist1 to suppress E-cadherin expression [331]. Notably, loss of FOXF2 expression promotes EMT in breast and liver cancers [332, 333]. In agreement, FOXF2 has shown to negatively regulate FOXC2-mediated cancer and EMT phenotypes [334]. This occurrence of closely linked downstream genes between FOXOs and other forkheads also highlights the involvement of tissue-specific misregulation or misexpression of FOX proteins, including FOXOs, during tumorigenesis, in particular in cancer metastasis.

## Targeting FOXO in cancer therapy

To date, many naturally derived and chemically synthesised compounds have been appraised for their potentials in cancer treatment. Remarkably, many compounds studied with demonstrated therapeutic capacities have been found to modulate FOXO expression and/or activity. This is due to the involvement of FOXO proteins in the regulation of genes linked to crucial drug action–related cellular processes, including stem cell renewal, DNA repair, cell death, metabolism, cell migration, angiogenesis, cell cycle control and cell division, as mentioned earlier. Therefore, not surprising, the mechanisms of resistance to conventional cytotoxic chemotherapeutics and to molecularly targeted therapies are also linked directly to deregulated signalling mediated through the FOXO transcription factors. Many currently used and proven cancer therapeutics, including paclitaxel, doxorubicin, epirubicin, lapatinib, gefitinib, imatinib and cisplatin, have been confirmed to mediate their cytotoxic and cytostatic functions through FOXO. For example, the anti-EGFR/HER small molecule inhibitors lapatinib and gefitinib have been shown to mediate their cytostatic and cytotoxic functions though activating FOXO3 *via* modulating the PI3K-Akt and p38/JNK/ERK pathways [31, 126, 127, 129, 131, 132]. Similarly, the therapeutic monoclonal antibody trastuzumab (Herceptin) directed against the extracellular domain of mutated HER2 also mediates its antiproliferative effects through FOXO3 and its downstream target survivin (BRC5) [335]. Genotoxic agents, including doxorubicin, epirubicin, cisplatin and 5-fluorouracil (5-FU), can also cause DNA damage and oxidative stress *via* FOXO3 [30]. The anthracycline doxorubicin induces FOXO3 activation and nuclear translocation by phosphorylating FOXO3 on Ser7 residue *via* p38 MAPK [336]. FOXO3 also induces doxorubicin-induced apoptosis through its transcriptional repression of miR-21 which, in turn, modulates the expression of pro-apoptotic factors, Fas-L, Bim and survivin [183, 337]. Another study shows that FOXO3 activation increases expression of TRAIL and cell death in response to doxorubicin in hepatocellular carcinoma (HCC), suggesting that FOXO3 is required for doxorubicin sensitivity. Conversely, FOXO3 inactivation and sequestration in the cytoplasm are closely associated with doxorubicin resistance [338]. Furthermore, SIRT1 has been shown to deacetylate p53 and FOXO3 which contributes to doxorubicin resistance [339]. In a similar manner, another anthracycline epirubicin, which is an epimer of doxorubicin, also mediates its anticancer activity through FOXO3 [200]. Other studies have verified FOXO3 as a key mediator of cisplatin action [182, 340]. Accordingly, it has been shown that cisplatin treatment causes a decrease in Akt-mediated FOXO3 phosphorylation in colon cancer cells, resulting in its nuclear translocation and activation [182]. Conversely, impaired FOXO3 nuclear accumulation and the consequent failure to induce apoptosis have been associated with the development of cisplatin resistance. Interestingly, increased FOXO3 activation in cisplatin-resistant colon cancer cells with the small molecule Akt inhibitor, tricirbine/API-2, can override cisplatin resistance by blocking Akt-mediated FOXO3 phosphorylation [182]. Paclitaxel functions by inducing microtubule dysfunction and, eventually, cell death by mitotic catastrophe [183, 184, 341, 342]. Paclitaxel has been shown to induce JNK-MAPK to phosphorylate and activate FOXO3 directly, as well as derepressing the inhibition of Akt on FOXO3 to induce apoptosis *via* Bim and other pro-apoptotic molecules [183, 184, 343]. Indeed, FOXO3 phosphorylation by p38/JNK MAPK on Ser7 can promote its nuclear localisation and activation as well as its acetylation by CBP/p300 on Lys-242/245 [180]. Compounds such as β-lapachone and 5-fluorouracil have been shown to induce cancer apoptosis through the activation of FOXOs and its target FOXM1 [344, 345].

Some naturally derived compounds including benzyl isothiocyanate, resveratrol, quercetin, arsenic trioxide and caffeic acid phenethyl ester (CAPE) have been shown to restrain cancer growth through FOXO activation [346]. Naturally derived compounds which can be found in daily diets also have a great potential in preventing cancer progression. Recently, studies with the extracted compounds are intensified to promote the concern for healthier diets. For instance, resveratrol in grapes promotes the transcriptional activity of FOXOs through AMPK to reduce ROS in a number of cancers [163]. Benzyl isothiocyanate (BITC) promotes the expression of FOXO-regulated Bim, p27^Kip1^ and p21^Cip1^ by reducing the Akt-mediated FOXO phosphorylation in pancreatic tumour [347]. Similarly, pterostilbene in blueberries activates FOXO1 by the suppression of Akt and ERK activity and increases 5-FU chemosensitivity in colon cancer [348]. A glutamate receptor antagonist, dizocilpine (also known as MK-801), has been shown to enhance FOXO1 nuclear localisation and promote its tumour suppressor function [349]. The chilli pepper extract capsaicin activates JNK to induce FOXO1 acetylation for Bim-induced apoptosis in pancreatic cancer [204]. Polydatin, a natural precursor of resveratrol, also upregulates FOXO1 through inhibition of Akt and STAT3 signalling pathway to suppress hepatocellular carcinoma cell migration and invasion [350]. Isorhapontigenin, another analogue of resveratrol, also inhibits STAT1 to enhance FOXO1 activity and limits bladder cancer cell invasion [351]. Polyphenol epigallocatechin-3-gallate in green tea can upregulate FOXO3 and suppress breast cancer metastasis [352]. Ergosterol from the mushroom *Amauroderma rude* also increases FOXO3 expression to inhibit cell migration and invasion [353]. Although some studies have exposed the underlying mechanisms of action of some of these compounds, the exact mechanisms for the rest of these naturally derived compounds are not fully understood. Thus far, none of these naturally derived compounds is officially used in the clinic for cancer treatment in consequence. Nevertheless, these findings further support the hypothesis that FOXOs serve as the sensor as well as the mediator of anticancer agents.

As FOXO expression and activity are often adaptively downregulated in malignancies and are further attenuated in drug-resistant cancer, the expression of some of their important downstream transcriptional targets is also diminished as a result. Thus, it is rational to propose that interventions aimed at the downstream targets of FOXO proteins could induce cancer and drug-resistant cell-specific elimination and form the basis for novel anticancer therapies [30]. Indeed, recent research has shown that PERK (PKR-like endoplasmic reticulum kinase, also called eukaryotic translation initiation factor 2-alpha kinase 3 (EIF2A3K)) is regulated by FOXO3 and thus exposed a transformed cell and chemotherapeutic drug–resistant cancer cell vulnerability in PERK [155]. In this case, small molecule PERK inhibitors, which have been generated and tested for neurodegenerative diseases, can be repositioned to target cancer cells.

## Conclusion

**Fig. 5:**
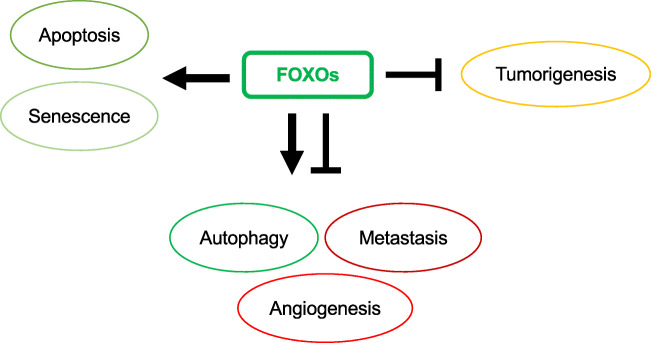
Role of the FOXO transcription factors. FOXOs exhibit tumour-suppressive roles in inhibiting tumorigenesis by promoting cell apoptosis and senescence. However, depending on the cell type and context–dependent manner, FOXOs may drive cell autophagy, metastasis and angiogenesis for the cell survival. Notably, FOXOs play a very important role in stress modulation and drug resistance

Collectively, research studies to date have pointed to a key tumour-suppressive role for FOXO transcription factors. This is mediated through the ability of FOXOs to regulate genes essential for cell proliferative arrest, cell death, autophagy, senescence, angiogenesis, cell migration and metastasis. FOXO proteins also integrate diverse proliferative, nutrient and stress signals with distinct gene networks to control cell fate, metabolism and cancer development. Notably, evidence also exists that FOXO proteins can sometimes support cancer progression, angiogenesis, metastasis and drug resistance in a cell type and context–dependent manner. Intriguingly, the mechanisms of action of conventional cytotoxic chemotherapeutics and novel molecularly targeted therapies are also invariably linked to FOXO transcription factors (Fig. 5). Thus, FOXO proteins have been firmly established as not only important markers for cancer diagnosis and prognosis but also targets for cancer intervention. Whilst the mechanisms underlying their roles and regulations are still being uncovered, accumulated research has also shown that FOXO expression and activity are predominantly regulated at the post-transcriptional and post-translational levels and are adaptively downregulated in cancer cells. Thus, targeting the upstream regulators of FOXOs may provide novel strategies of cancer treatment and for overcoming drug resistance. Equally, the FOXO downstream targets, which are adaptively downregulated with FOXOs in cancer, may represent vulnerabilities for cancer intervention strategies.
